# Host-free biofilm culture of “*Candidatus* Liberibacter asiaticus,” the bacterium associated with Huanglongbing

**DOI:** 10.1016/j.bioflm.2019.100005

**Published:** 2019-09-11

**Authors:** Phuc T. Ha, Ruifeng He, Nabil Killiny, Judith K. Brown, Anders Omsland, David R. Gang, Haluk Beyenal

**Affiliations:** a Gene and Linda Voiland School of Chemical Engineering and Bioengineering, Washington State University, Pullman, WA, 99163, USA; b Institute of Biological Chemistry, Washington State University, Pullman, WA, 99163, USA; c Department of Plant Pathology, Citrus Research and Education Center, University of Florida, Lake Alfred, FL, 33850, USA; d School of Plant Sciences, University of Arizona, Tucson, AZ, 85721, USA; e Paul G. Allen School for Global Animal Health, Washington State University, Pullman, WA, 99163, USA

**Keywords:** Biofilm, Host-free culture, Isolation, *Candidatus* Liberibacter asiaticus, Huanglongbing

## Abstract

Inability to culture the phloem-restricted alpha-proteobacterium “*Candidatus* Liberibacter asiaticus” (“*Ca.* L. asiaticus”) or the closely related species (“*Candidatus* Liberibacter americanus” and “*Candidatus* Liberibacter africanus”) that are associated with Huanglongbing (HLB) hampers the development of effective long-term control strategies for this devastating disease. Here we report successful establishment and long-term maintenance of host-free “*Ca.* L. asiaticus” cultures, with the bacterium growing within cultured biofilms derived from infected citrus tissue. The biofilms were grown in a newly designed growth medium under specific conditions. The initial biofilm-based culture has been successfully maintained for over two years and has undergone over a dozen subcultures. Multiple independent cultures have been established and maintained in a biofilm reactor system, opening the door to the development of pure culture of *“Ca.* L. asiaticus*”* and the use of genetics-based methods to understand and mitigate the spread of HLB*.*

## Introduction

Huanglongbing (HLB), also known as citrus greening disease, is a threat to worldwide citrus production, a multibillion dollar industry. Diagnostic tests have consistently shown that citrus trees displaying symptoms of HLB in the USA are infected with “*Candidatus* Liberibacter asiaticus” (“*Ca.* L. asiaticus”), a phloem-restricted alpha-proteobacterium that is spread by the Asian citrus psyllid (*Diaphorina citri*, ACP) [[1], [2], [3], [4]]. The fastidious nature of “*Candidatus* Liberibacter sp.” has limited our ability to grow these pathogens in axenic culture, which in turn has hampered studies of the etiology, mechanism, and control of HLB. Inability to culture this presumptive causative agent of HLB *in vitro* also prevents the fulfillment of Koch's postulates to confirm disease causality. Several attempts have been made to culture “*Ca.* L. asiaticus,” but none have entirely succeeded. Moreover, “*Ca.* L. asiaticus” has only been reported to be maintained in short-term cultures (e.g., no repetitive transfers or long-term maintenance) [[5], [6], [7]]. Because of the limited success in previous work as detailed below, there is a need for tools for reproducible and long-term culture of host-free “*Ca.* L. asiaticus.”

A decade ago, Sechler et al. [6] claimed successful cultivation of “*Ca.* L. asiaticus” in which pure cultures of “*Ca.* Liberibacter spp*.*” were obtained using Liber A medium made of citrus vein extract. To the best of our knowledge, there were no follow-up or independent repetitions, either within the group or by others. In fact, there is still no pure culture of “*Ca.* L. asiaticus” that researchers can access or reproduce. These problems suggest the difficulty associated with the long-term reproducibility of culturing methods for “*Ca.* Liberibacter spp*.*” Another attempt to isolate “*Ca.* L. asiaticus” *in vitro* resulted in an initial co-culture of “*Ca.* L. asiaticus” with an Actinobacterium species [5]. Although this seems to be a promising finding, unfortunately there was limited evidence of the long-term reproducible growth of “*Ca.* L. asiaticus” with Actinobacterium. A later attempt to prolong the viability of “*Ca.* L. asiaticus” *in vitro* by adding citrus juice to King's B culture medium resulted in a mixed culture containing “*Ca.* L. asiaticus” and other dominant bacterial genera [7]. However, “*Ca.* L. asiaticus” was detected in the cultures of only certain inoculation attempts (e.g., no continuous transfer or long-term maintenance). It is striking that of the 17 experiments which were initiated with the inoculum with the extract from citrus seed coat, only 4 with the highest concentration of “*Ca.* L. asiaticus” were selected for qPCR analysis. It is possible that “*Ca.* L. asiaticus” survived in those cultures because of available nutrients remaining from the inoculum. Fujiwara et al. recently reported the growth of “*Ca.* L. asiaticus” strain Ishi-1 on a solid medium in association with microbiota originating from citrus phloem [8]. Disruption of the microbiota using an antibiotic hindered the survival of “*Ca.* L. asiaticus” Ishi-1, suggesting that cohabiting bacteria are important for “*Ca.* L. asiaticus” to survive. However, the medium used for testing the *in vitro* survival of “*Ca.* L. asiaticus” Ishi-1 failed to culture other “*Ca.* L. asiaticus” strains, including Miyako-13. It should be noted that the Ishi-1 strain is unique and has a genome size of 1.19 Mb [9]. Most other “*Ca.* L. asiaticus” genomes sequenced, including strains circulating in the United States and Miyako-13, are larger (∼1.23 Mb) and contain prophage sequences, which Ishi-1 does not [9]. Although they had limited success with other strains, Fujiwara et al.‘s work suggests that “*Ca.* L. asiaticus” growing *in vitro* may need to obtain essential nutrients through a mutualistic relationship with other organisms.

Recent studies on culturing other fastidious organisms suggest that these organisms require specific nutrients, pH, incubation temperatures and/or oxygen levels that are not compatible with conventional aerobic culture methods [3,10,11]. “*Ca.* L. asiaticus” multiplies in the phloem sap of citrus plants and in the haemolymph/gut of the Asian citrus psyllid (ACP), its primary insect vector [2,3,12,13]. These are two distinct niches with unique nutrients and/or physicochemical conditions that are essential for pathogen growth. These unique environments that support “*Ca.* L. asiaticus” growth may depend, in part, on the presence of other microorganisms/endosymbionts. Plant phloem sap contains a large variety of sugars, amino acids, organic acids, vitamins, and inorganic ions [3,17], many of which are also present in ACP haemolymph [18]. Genome assembly and annotation from the metagenomics sequence data available for the various “*Ca.* Liberibacter” species established that they all lack genes coding for essential enzymes and other proteins [[14], [15], [16]]. We hypothesized that “*Ca.* L. asiaticus” can grow in biofilm and that this biofilm culture can be replicated for long-term host-free “*Ca.* L. asiaticus” growth.

To test our hypothesis, we designed a membrane biofilm reactor (MBR) and inoculated it with the extract from “*Ca.* L. asiaticus”-infected Hamlin sweet orange leaf and stems. We used a new medium with a high-strength (neutral pH) buffer along with nutrients including amino acids, trace minerals and vitamins. We showed that this combined system is able to support long-term growth and repeated sub-culturing of “*Ca.* L. asiaticus” together with other microorganisms in a mixed-community biofilm. We observed consistent presence and growth of “*Ca.* L. asiaticus” in biofilm cultures even after a dozen transfers spanning over two years. Multiple independent cultures have been established and maintained in the biofilm reactor system. To the best of our knowledge, this is the first successful establishment of a host-free culture of “*Ca.* L. asiaticus.”

## Materials and methods

### Membrane biofilm reactor: construction and operation

**Fig. 1:**
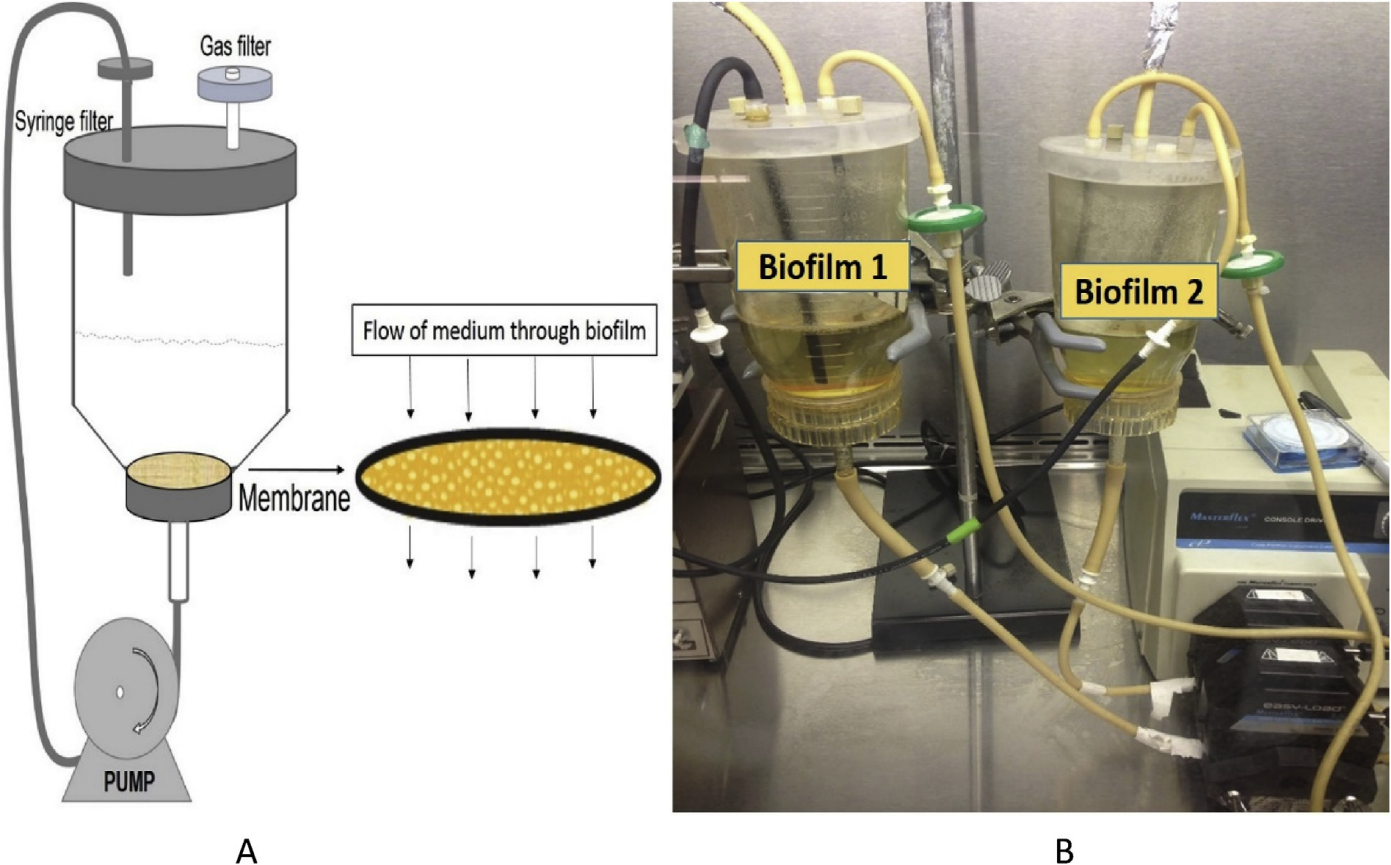
(a) Schematic diagram of the membrane biofilm reactor used to culture “*Ca*. L. asiaticus.” (b) Photo of the actual membrane biofilm reactors used to develop initial “*Ca*. L. asiaticus” biofilms through inoculation with extract from midribs of leaves and stems of “*Ca*. L. asiaticus”-infected citrus plants.

Biofilms were grown using a custom-built membrane biofilm reactor (MBR) (Fig. 1). The reactor was built using a filter funnel from Millipore (Millipore, USA) and a polyvinylidene fluoride membrane filter with a pore size of 0.1 μm (Millipore). The reactor and tubing were sterilized by autoclaving, and the filter membrane was sterilized by exposure to UV radiation (27 kJ, 30 min per side). After sterilization, the membrane was placed and secured in the funnel. Next, 150 ml of medium was pumped into the filter funnel. The reactor funnel was then inoculated with inoculum containing “*Ca.* L. asiaticus.” Biofilms were grown for 10–15 days at room temperature. Oxygen was delivered by sparging the liquid medium with the appropriate gas (100% N_2_ for anaerobic condition, 90% N_2_:10% O_2_ for 10% oxygen tension, 100% air for aeration).

### Medium composition and preparation

Our new growth medium for “*Ca.* L. asiaticus” biofilm culture was based on BM7 medium, which is used to isolate and grow *L. crescens* [16], with several modifications. First, we lowered the nutrient concentrations by diluting the BM7 medium; second, we increased the buffering capacity; and third, the medium was enhanced with a complex mixture of salts, vitamins and trace minerals (Table 1 and Table 2).

**Table 1:** Composition of growth medium (1 L) for “*Ca.* L. asiaticus” biofilm culture.

INGREDIENT	AMOUNT
Alpha-ketoglutaric acid	2 g
ACE buffer	10 g
KOH	3.75 g
Salt mixture (100X)	10 ml
Phosphate buffer, 1 M (pH 7.0)	10 mL
DI water	Adjust to 880 mL
Adjust the pH of above solution to 7.0 and autoclave at 121 °C. After cooling, aseptically add:	
Fetal bovine serum	25 mL
Hink's TNM-FH insect medium	75 mL
Vitamin mixture (100X)	10 mL
Trace mineral mixture (100X)	10 mL

**Table 2:** Composition of salt, trace mineral and vitamin mixtures used in “*Ca.* L. asiaticus” growth medium.

**100 × Salt mixture**	**Concentration (g/L)**
KCl	38.0
NH_4_Cl	20.0
NaH_2_PO_4_ · H_2_O	6.9
CaCl_2_ · 2H_2_O	4.0
MgSO_4_ · 7H_2_O	20.0
**100 × Trace mineral mixture**	**g/L**
Nitrilotriacetic acid (NTA)	1.500
- pH NTA solution to 10, then add:	
MnCl_2_ · 4H_2_O	0.100
FeSO_4_ · 7H_2_O	0.300
CoCl_2_ · 6H_2_O	0.170
ZnCl_2_	0.100
CuSO_4_ · 5H_2_O	0.040
AlK(SO_4_)_2_· 12H_2_O	0.005
H_3_BO_3_	0.005
Na_2_MoO_4_	0.090
NiCl_2_	0.120
NaWO_4_· 2H_2_O	0.020
Na_2_SeO_4_	0.100
- pH solution to 7.0	
**100 × Vitamin mix**	**mg/L**
Biotin	2.0
Folic acid	2.0
Pyridoxine hydrochloride	10.0
Riboflavin	5.0
Thiamine hydrochloride	5.0
Nicotinic acid	5.0
*DL*-calcium pantothenate	5.0
Vitamin B_12_	0.1
*p*-Aminobenzoic acid	5.0
Thiocidic (lipoic) acid	5.0

### Inoculum preparation

The biofilm culture of “*Ca.* L. asiaticus” was initiated from citrus plant extracts. “*Ca.* L. asiaticus”-infected Hamlin sweet orange leaf and stem samples were prepared at the University of Florida Citrus Research and Education Center and shipped overnight at ambient temperature to Washington State University, Pullman, WA. Upon arrival at WSU, the citrus plant samples were used immediately for inoculum preparation or, if this was not possible, they were kept at 4 °C for no more than 2 days until use. The leaves and stems were first washed with deionized water to remove large particles, followed by a surface disinfection procedure. Surface disinfection was carried out in a laminar flow cabinet by immersing leaves and stems sequentially in 10% bleach, 5% bleach and 75% ethanol. Each step was conducted for 10 min. After this, the leaves and stems were rinsed several times with autoclaved distilled water to remove bleach and ethanol. Leaf mid-ribs were collected using sterilized razor blades. Mid-ribs and stems were cut into small pieces (∼0.5 cm) in sterilized petri dishes (ThermoFisher) before being crushed gently with a mortar and pestle at room temperature. The crushed/blended midribs and stems were transferred via scraping with a sterilized spatula to a sterile Falcon tube containing 10 mL of fresh culture medium. The mixture was vortexed vigorously at maximum speed for 5 min before being centrifuged at 2500 *g* for 5 min. The supernatant was carefully transferred to a sterile Falcon tube and used as an inoculum for membrane biofilm reactors (MBRs).

Biofilm was allowed to grow in the inoculated MBR for 10–15 days. For repeated sub-culturing, a new sequential biofilm culture was generated by transferring a small fraction of this homogenized mixture of biofilm and planktonic culture to sterile MBRs with fresh medium (∼1% inoculated volume). Between transfers and during long-term maintenance, the mixture of previous biofilms and planktonic culture was kept in a fridge (4 °C). Control reactors were operated using either extracts from “*Ca.* L. asiaticus”-free citrus plants or autoclaved biofilm culture (at 15 psi, 121 °C for 15 min) as the inactivated inoculum.

### Biofilm culture sampling

Biofilm cultures from MBRs were sampled at the time of the initial inoculation and at the end of biofilm growth (∼10–15 days). At the initial inoculation, the inoculum was mixed with the medium and a small amount was collected via the sampling outlet of the MBR using a sterile syringe. Samples for PCR or qPCR were aliquoted and kept at −20 °C until DNA was extracted for analysis. At the end of each transfer (∼10–15 days), the upper part of the planktonic culture was gently poured into a sterile conical tube. Next, the filter funnel was disassembled. Membranes harboring biofilm were carefully transferred to sterile, new petri dishes using sterilized tweezers. All steps were carried out aseptically in a biosafety cabinet. The biofilm-supporting membrane was cut into portions for further experiments such as scanning electron microscopy and genomic DNA extraction. The rest of the biofilm was mixed with the planktonic culture (in the conical tube) and homogenized by vigorous vortexing.

To determine the growth of “*Ca.* L. asiaticus” in MBRs over time, several MBRs were inoculated using the same inoculum and operated under the same conditions. The biofilm cultures from these MBRs were then sampled sequentially at several points in time (e.g., at days 5, 7, 10, and 15 after culture initiation).

### Genomic DNA extraction, PCR and qPCR analysis

Genomic DNA (gDNA) was extracted from the initial citrus plants and from biofilm culture samples. Plant DNA was extracted using the Plant DNEasy Kit (QIAGEN, Germany) following the manufacturer's instructions. Community genomic DNA from biofilm samples was extracted using a manual extraction method. Briefly, each biofilm culture sample was centrifuged (17,000 *g*, 10 min) and the pellet was washed twice with 500 μL of the washing buffer (10 mM Tris–HCl pH 8.0, 1 mM EDTA and 100 mM NaCl) and then resuspended with 300 μL of Tris-EDTA (TE) buffer (10 mM Tris–HCl pH 8.0, 1 mM EDTA). Samples were then transferred to Lysis matrix E tubes (MP Biomedicals, USA), fixed onto a flat-vortexer (VWR, USA) and beaded for 4 min at maximum speed. After a brief centrifugation (16,000 *g*, 90 s), the supernatant was recovered into a new microcentrifuge tube. Sodium dodecyl sulfate (SDS, 10%) and Proteinase K (Fisher Scientific, USA) were added to final concentrations of 1% and 0.2 mg/mL, respectively, prior to incubation at 56 °C for 1 h. Post lysis, DNA was extracted with phenol-chloroform-isoamyl alcohol (25:24:1, v:v:v) and then chloroform-isoamyl alcohol (24:1, v:v). The DNA was then precipitated by adding 0.1 vol of 5 M sodium acetate (pH 5.5) and 2 vol of 100% cold ethanol and keeping the sample at −80 °C for 30 min. DNA was collected by centrifugation (16,000 *g*, 10 min), washed in 70% ethanol, dried, and resuspended in TE buffer.

Several sets of primers that are specific for “*Ca.* L. asiaticus” were used throughout this study. For PCR, we used two primer sets specific to 16S rDNA fragments of “*Ca.* L. asiaticus,” (Ol1/Ol2c) [19] and (Las606/LSS) [20], to determine the presence of the bacterium in the extracted gDNA samples using PCR. PCR was performed in 20-μL reactions containing 1 μL of genomic DNA (extracted as described above), 1 × PCR buffer, 1.5 mM MgCl_2_, 0.1 μM of each primer (IDT), 0.4 μL of 10 mM dNTPs and 1.00 unit of Platinum *Taq* DNA Polymerase (Invitrogen, USA). PCR was performed using a Mastercycler® thermal cycler (Eppendorf, USA) under the following conditions: (i) an initial denaturation step at 95 °C for 3 min, (ii) 35 amplification cycles (94 °C for 45 s, 58 °C for 30 s and 72 °C for 90 s), and (iii) a final extension at 72 °C for 5 min.

For qPCR, we designed primers specific to “*Ca.* L. asiaticus” 16S rDNA fragments (forward primer, Las1F: 5′-GGTTTTTACCTAGATGTTGGGTACT-3′; reverse primer, Las1R: 5′-CTTCGCAACCCATTGTAACC-3′) to quantify the 16S rDNA gene of “*Ca.* L. asiaticus” in the gDNA samples using qPCR. This primer set is only present in the “*Ca.* L. asiaticus” 16S rDNA gene, and it amplifies a 140-bp fragment specific to “*Ca.* L. asiaticus.” The qPCR reactions were performed using the Step One Real-Time PCR system (Applied Biosystems, USA) as follows: 3 min of incubation at 95 °C; then, 40 cycles of 15 s at 95 °C, 15 s at 57 °C and 20 s at 68 °C. Fluorescent signals were collected at the 68 °C stage of each cycle. The 20-μL qPCR reaction mixture contained 10 μl of 2 × PerfeCTa® SYBR® Green SuperMix (Quantabio, USA), 1 μl of DNA template, 0.1 μM of each primer (IDT), and Nuclease-Free Water (ThemoFisher, USA). All reactions were performed at least with triplicate technical replicates, and each run contained negative control samples (H_2_O) and positive control samples (gDNA from “*Ca.* L. asiaticus”-infected plants). The qPCR data were analyzed with the Applied Biosystems software (version 2.3). Since each genome of “*Ca.* L. asiaticus” contains 3 copies of the 16S rDNA gene [21], the qPCR results were converted to the number of “*Ca.* L. asiaticus” genome equivalents using a standard curve created using plasmids containing the 140-bp fragment that had been amplified from “*Ca.* L. asiaticus” using Las1R and Las1F and cloned into the pCR™4-TOPO^TM^ plasmid. Most of the samples from initial cultures (inoculation time, day 0) did not show detectable amplification of qPCR product specific to “*Ca.* L. asiaticus” in chromatograms. In this case, we used the cycle threshold (Ct) value of 40 (Ct = 40) to calculate the number of genome equivalents (GEs) in these samples. We should note that we detected “*Ca.* L. asiaticus” in our original source. However, when it was used to inoculate a reactor, it needed to be diluted (1 mL source/150 mL growth medium). Due to dilution, we could not detect “*Ca.* L. asiaticus” which practically meant that this was below our detection limit.

Another set of primers specific to the conserved “*Ca*. L. asiaticus” hypothetical gene CD16-00155 (Forward: 5′-TCTAAAGCAGGACGTTTGTGT-3′; Reverse: 5′-TGAGTATATTTGATTCTCGTGCAA-3′) was used as the second confirmation of the presence and growth of “*Ca*. L. asiaticus.” For this primer set, the qPCR reactions were performed as follows: 5 min of incubation at 95 °C; then, 40 cycles of 15 s at 95 °C, 30 s at 62 °C and 30 s at 72 °C.

### Cloning and sequencing

Clone libraries were constructed in order to determine the specificity of the DNA fragments amplified by Ol1/Ol2c (1160 bp) and Las1R/Las1F (140 bp). PCR and qPCR products extracted from biofilm cultures at the 3rd, 5th and 12th transfers using a gDNA template were gel-purified using the QIAquick Gel Extraction Kit (QIAGEN, Germany) and cloned into pCR™4-TOPO^TM^ vector (Invitrogen™), followed by transformation into One Shot™ TOP10 competent *Escherichia coli* cells (Invitrogen™), using the manufacturer's protocols. Plasmids containing the insert were sent for sequencing (WSU Genomics Core, USA). Sequences were analyzed using MEGA 7.0 and compared to known sequences in GenBank using the Basic Local Alignment Search Tool algorithm against the nucleotide collection database (BLASTn) on the National Center for Biotechnology Information (NCBI) website. Sequences were deposited in NCBI's GeneBank database under submission number SUB4773845. A phylogenetic tree was constructed in MEGA 7.0 [22] using the built-in neighbor-joining (NJ) method [23], with evolutionary distance calculated by maximum composite likelihood, and using the pairwise deletion option and bootstrapping with 1000 replicates.

### Community analysis

For the microbial community analysis of the biofilm cultures, 250 μL of each culture was sampled and homogenized for gDNA extraction. The extraction was performed using a QIAGEN MagAttract PowerMicrobiome Kit (QIAGEN, USA). After extraction, samples were quantified using the Quant-iT PicoGreen dsDNA Assay Kit (Invitrogen, USA). The DNA extraction and libraries were prepared by the University of Michigan Host Microbiome Core as described previously [24]. Briefly, the V4 region of the 16s rDNA gene was amplified from each sample using the dual indexing sequencing strategy developed by Kozich et al. [25] The PCR reactions were composed and performed as per an established protocol [24].

Amplicon samples were normalized using a SequalPrep Normalization Plate Kit (Life Technologies) following the manufacturer's protocol for sequential elution. Samples were pooled and the concentration of the pooled samples was determined using the Kapa Biosystems Library Quantification Kit for Illumina platforms (KapaBiosystems). The sizes of the amplicons in the library were determined using the Agilent Bioanalyzer High Sensitivity DNA Analysis Kit (Agilent). Libraries and sequencing reagents were prepared according to Illumina's protocols (“Preparing Libraries for Sequencing on the MiSeq” and “16S Sequencing with the Illumina MiSeq Personal Sequencer”) as described previously [24]. Amplicons were sequenced on the Illumina MiSeq platform using a MiSeq Reagent 222 Kit V2 for 500 cycles according to the manufacturer's instructions with modifications for the primer set. FASTQ files were generated for paired-end reads.

### Analysis of microbiota community

Raw sequence files (FASTQ files) were deposited in the NCBI's Sequence Read Archive (SRA) database under submission number SUB4773868. Sequences were analyzed using mothur v.1.39 [26] according to the MiSeq standard operating procedure (http://mothur.org/wiki/MiSeq_SOP) using the newest SILVA reference (SILVA v132) [27]. Briefly, filtered sequences were dereplicated and aligned to the newest SILVA-based reference alignment (silva.nr_v132.align). The sequences were then screened to remove those that did not align to positions 11894–25319 of the reference alignment, filtered to remove non-informative columns, preclustered to >99.0% identity (allowing 2 differences), and dereplicated. Chimeras were identified and removed using UCHIME as implemented in mothur v.1.39 in self-referential mode. Filtered sequences were classified against the SILVA (v132) reference taxonomies using a naive Bayesian classifier implemented within mothur [28] with an 80% bootstrap cutoff, and sequences that were not bacteria were removed using remove.lineage. Operational taxonomic units (OTU) were identified using a 97% similarity rate and used for downstream community analyses. OTUs were classified based upon the sequence classifications described above.

### Scanning electron microscopy

Filter membranes with biofilms formed on them were imaged using scanning electron microscopy (SEM). Samples were fixed with a 0.1 M phosphate-buffered saline (PBS) solution containing 2.0% glutaraldehyde and 2.0% paraformaldehyde, at pH 7.2, for up to 12 h at 4 °C, then washed 3 times in 0.1 M PBS (pH 7.2) for 10 min each time, and then dehydrated further in ethanol solutions (in sterile water) of 10%, 35%, 50%, 75%, 95% and 100% for 10 min in each solution. The dehydration step in 100% ethanol (200 proof) was performed 3 times. The samples were immediately immersed twice, for 10 min each time, in hexamethyldisilazane (Sigma-Aldrich, MO, USA), followed by air-drying for 9 h in a fume hood. The samples were sputter-coated with gold and were imaged with a Quanta SEM (FEI, TX, USA).

## Results and discussion

### Growth of “*Ca.* L. asiaticus” in membrane biofilm reactors

**Fig. 2:**
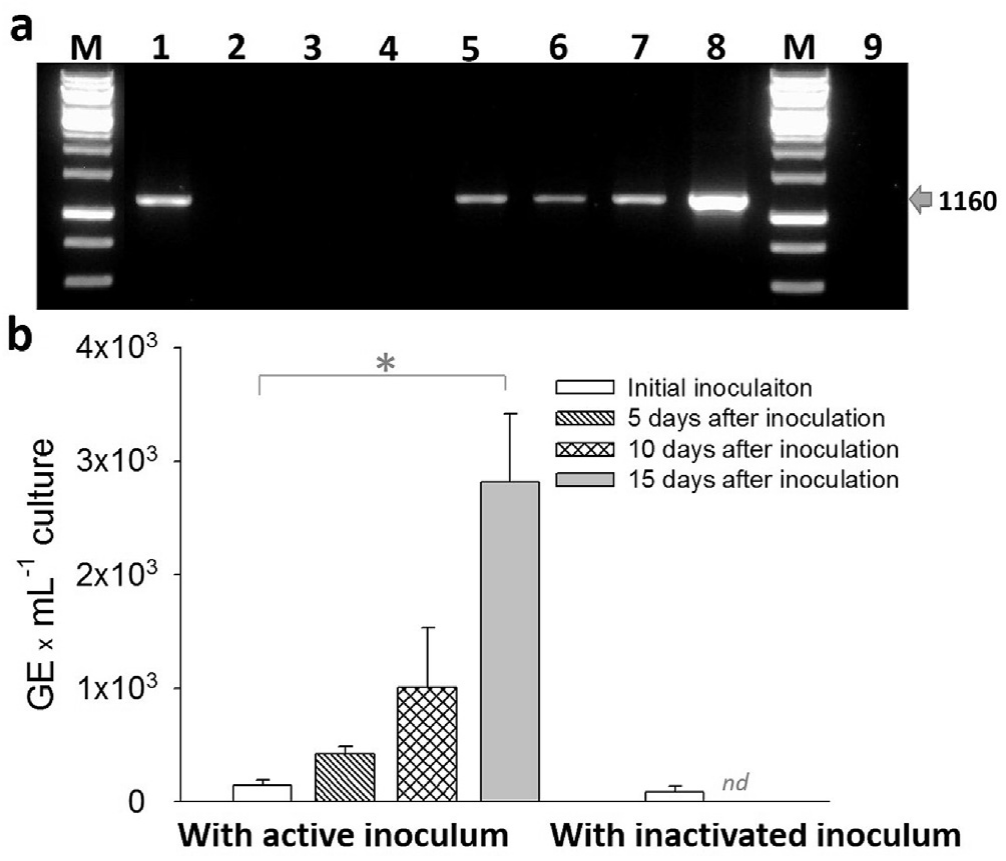
Growth of “*Ca.* L. asiaticus” in biofilm cultures. Biofilms were cultured in the MBRs without continuous aeration at pH 7.0. **a)** PCR amplification of a 1160-bp fragment of the “*Ca.* L. asiaticus” 16S rDNA gene using specific primers Ol1 and Ol2c. Lanes marked M contained a 1-kb ladder; lanes 1 and 8: infected citrus Hamlin; lane 2: initial inoculation with inactivated inoculum; lane 3: 15 days after inoculation with inactivated inoculum; lane 4: initial inoculation with 1% of active inoculum from 2^nd^ transfer; lane 5: biofilm 10 days after inoculation with active inoculum; lane 6: planktonic culture 10 days after inoculation with active inoculum; lane 7: mixture of biofilm and planktonic cultures 10 days after inoculation with active inoculum; lane 9: water. **b)** Validation and quantification of “*Ca.* L. asiaticus” growth in the MBR at the 5th transfer using qPCR with a probe specific for the 16S rDNA of “*Ca.* L. asiaticus.” The error bars represent the standard error of the mean of independent experiments (*n* = 4). An asterisk indicates a significant difference (one-way ANOVA test, P« 0.05) among the tested samples. The notation “*n/d*” indicates there was no detectable signal in qPCR.

The growth in each subsequent culture in the MBRs with our medium was detected using PCR with conventional primers (Ol1/Ol2c) (Fig. 2a) and monitored using quantitative real-time PCR (qPCR) analysis (Fig. 2b). Traditional PCR generated a 1160-bp fragment that was below the limit of detection in the inoculum under the PCR conditions used when monitored by gel electrophoresis (Lane 2, Fig. 2a) but was amplified and readily detected in this analytical system from biofilm cultures after 5, 10 and 15 days of MBR operation. “*Ca.* L. asiaticus” GEs were quantified during each transfer using qPCR analysis. Fig. 2b shows the change in “*Ca.* L. asiaticus” GEs in the biofilm culture at the 5th sequential subculture after 5, 10 and 15 days of MBR operation, suggesting growth of “*Ca.* L. asiaticus” over time. Other sub-cultures (more than 12 transfers over more than two years) and independently initiated cultures displayed similar growth patterns (SI Fig. 1, SI Fig. 2). *“Ca*. L. asiaticus” was also detectable in PCR and qPCR assays using different primers targeting alternative sequences (SI Fig. 2). Further cloning and sequencing of the 1160-bp DNA fragments amplified by PCR confirmed specificity to “*Ca.* L. asiaticus,” with >99% identity to “*Ca.* L. asiaticus” 16S rDNA (29 of 29 sequences from biofilm and planktonic cultures, SI Fig. 3). Cloning and sequencing of the 140-bp fragment amplified from qPCR products also confirmed their specificity to “*Ca.* L. asiaticus,” with 100% identity to “*Ca.* L. asiaticus” 16S rDNA (27/27 sequences from biofilm culture at the 5th transfer, 18/18 sequences from biofilm culture at the 12th transfer).

**Fig. 3:**
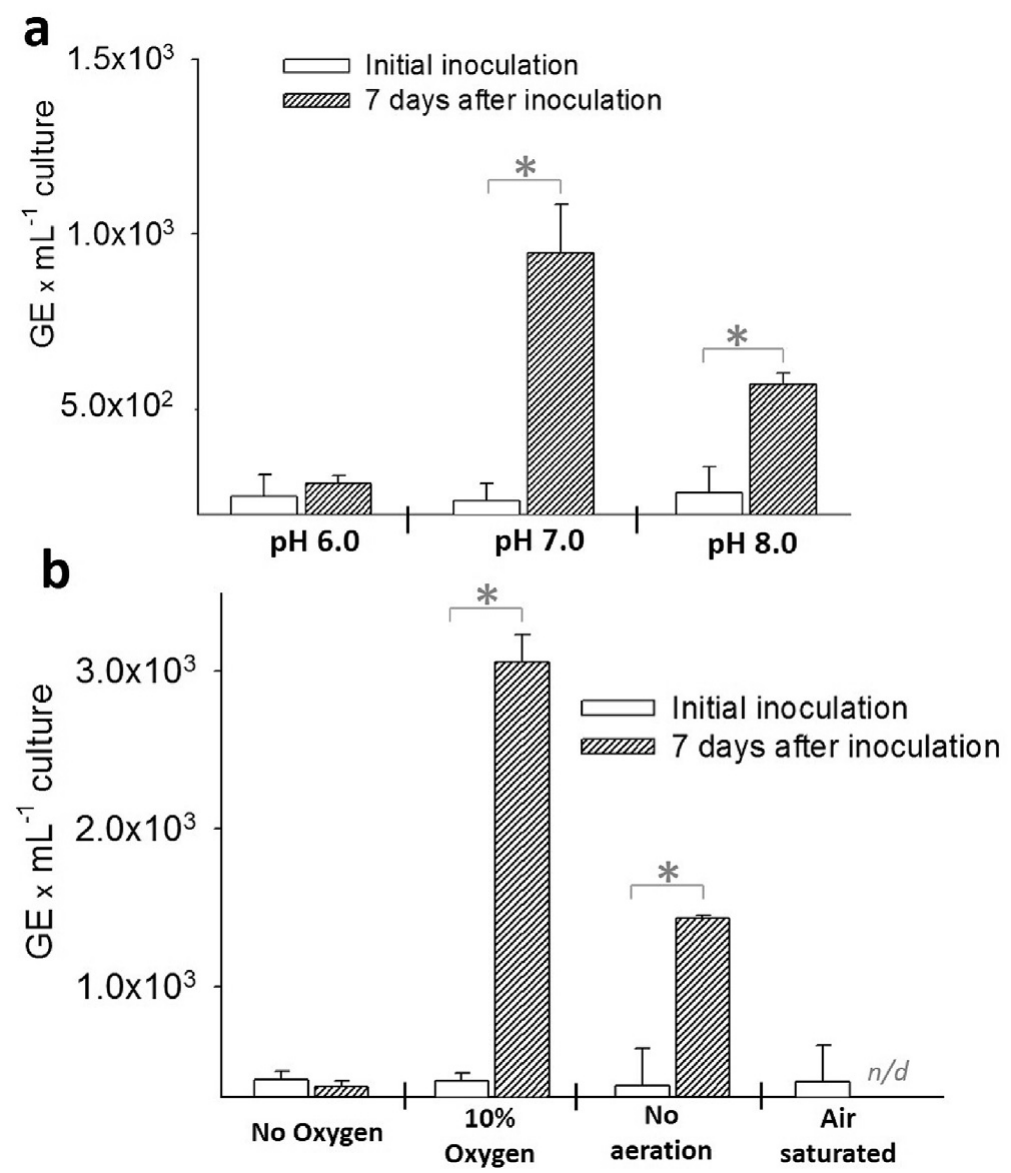
Effects of pH and oxygen tension on the growth of “*Ca.* L. asiaticus” in a mixed culture with other biofilm microorganisms. **a)** No significant growth was detected at pH 6, but there was growth at pH 7 and 8. All cultures were grown without aeration. **b)** Higher GE levels of “*Ca.* L. asiaticus” were measured in the cultures grown under 10% oxygen tension than in those grown under other oxygen tensions. No growth was detected in the culture purged with N_2_ or in the culture with an oxygen-saturated condition. All cultures were grown at pH 7. The error bars represent the standard error of the mean of replicated experiments (*n* = 3). An asterisk indicates a significant difference (one-way ANOVA test, P« 0.05) among the tested samples. The notation “*n/d*” indicates there was no detectable (or below detection limit) signal in qPCR.

To verify the success of our biofilm culturing method, we examined the growth of “*Ca.* L. asiaticus” in biofilm cultures over multiple transfers and repetitions, including independent transfers in different laboratories (housed in different buildings). Over the course of more than two years, we have transferred the initial biofilm culture over a dozen times (∼10–15 days of MBR operation per subculture). Between transfers, the cultures were maintained at 4 °C until verified by qPCR for “*Ca.* L. asiaticus” growth and the MBR was cleaned, sterilized and prepared for the next transfer. The PCR and qPCR results shown in SI Fig. 1a and b and SI Fig. 2 demonstrate the presence and growth of “*Ca.* L. asiaticus” during the 7th to 9th and the 12th transfers, which were conducted independently in a different laboratory in the Institute of Biological Chemistry (Gang lab) using the original inoculum from the 6th transfer generated in the School of Chemical Engineering and Bioengineering laboratory (Beyenal lab). That the culture could be stored and used by different researchers at a later date to reinitiate sequential cultures confirms the stability and transferability of “*Ca.* L. asiaticus”-containing biofilms cultured using our methodology. Independently, we also successfully repeated the MBR experiment using new extracts from “*Ca.* L. asiaticus”-infected citrus plants as the inoculum. We repeated this experiment another three times using citrus plant samples collected at different times. Growth of “*Ca.* L. asiaticus” in biofilm culture was observed in all independent experiment, and data from a representative experiment is presented in SI Fig. 1c and d. These results confirm the reproducibility of our host-free “*Ca.* L. asiaticus”- culturing approach using the newly designed medium.

Together, these results demonstrate our ability to grow “*Ca.* L. asiaticus” in axenic culture in biofilm form by inoculating from “*Ca.* L. asiaticus” -infected citrus plants. Previous reports describing the successful culturing of “*Ca.* L. asiaticus” lacked strong evidence for the long-term growth of “*Ca.* L. asiaticus” [3,10,11]. In addition, their cultures were not tested for subsequent transfers or independently reproduced at different times. In fact, in one of the most recent culturing efforts, Parker et al. [7] reported the detection of “*Ca.* L. asiaticus” within a mixed culture during a single experiment (i.e., no sub-sequential transfers); this can be explained as just prolonged viability due to citrus juice (e.g., antioxidant) addition. In our biofilm reactor experiments, “*Ca.* L. asiaticus” clearly grew substantially after only 5–15 days of growth, which could be repeated over and over again. Thus, our newly designed medium and the MBRs used in this study allowed “*Ca.* L. asiaticus” to grow and form interactions with other microorganisms that appear to support maintenance of “*Ca.* L. asiaticus” and its growth *in vitro*. “*Ca.* L. asiaticus” was detected not only in the biofilms, but also in the planktonic phase of the MBRs (Fig. 2a), suggesting that either “*Ca.* L. asiaticus” can grow in the planktonic phase as long as appropriate nutrients are present or bacteria from the growing biofilms are sloughed off during the culture period. Nutrients in the planktonic phase may come from the nutrient medium or from mutualistic bacteria in the biofilm because the medium in MBRs continuously recirculates, allowing nutrients released from the biofilm back into the bulk medium.

### “*Ca.* L. asiaticus” prefers neutral to alkaline pH and low oxygen tension

“*Ca.* L. asiaticus” was found to prefer neutral to alkaline pH (pH 7.0–8.0) and low oxygen tension (∼10%) over over anaerobic conditions (Fig. 3). These findings agree with results from our recent work, in which pH and oxygen tension inside ACP abdomens were measured using microelectrodes (unpublished data). Interestingly, while a high-density “creamy-orange” culture was obtained when oxygen was available (OD_600_ reached 1.6–1.8 after two weeks for 10% oxygen), we observed only light growth of a creamy-white culture with significantly reduced numbers of “*Ca.* L. asiaticus” GE under anaerobic conditions (OD_600_ reached only 0.3). These results suggest further that either “*Ca.* L. asiaticus” is aerobic, requiring oxygen for growth, or oxygen is required for the growth of specific microorganisms that are crucial for the growth of “*Ca.* L. asiaticus” in mixed culture.

### Growth of “*Ca.* L. asiaticus” *in vitro* in association with its microbiota

**Fig. 4:**
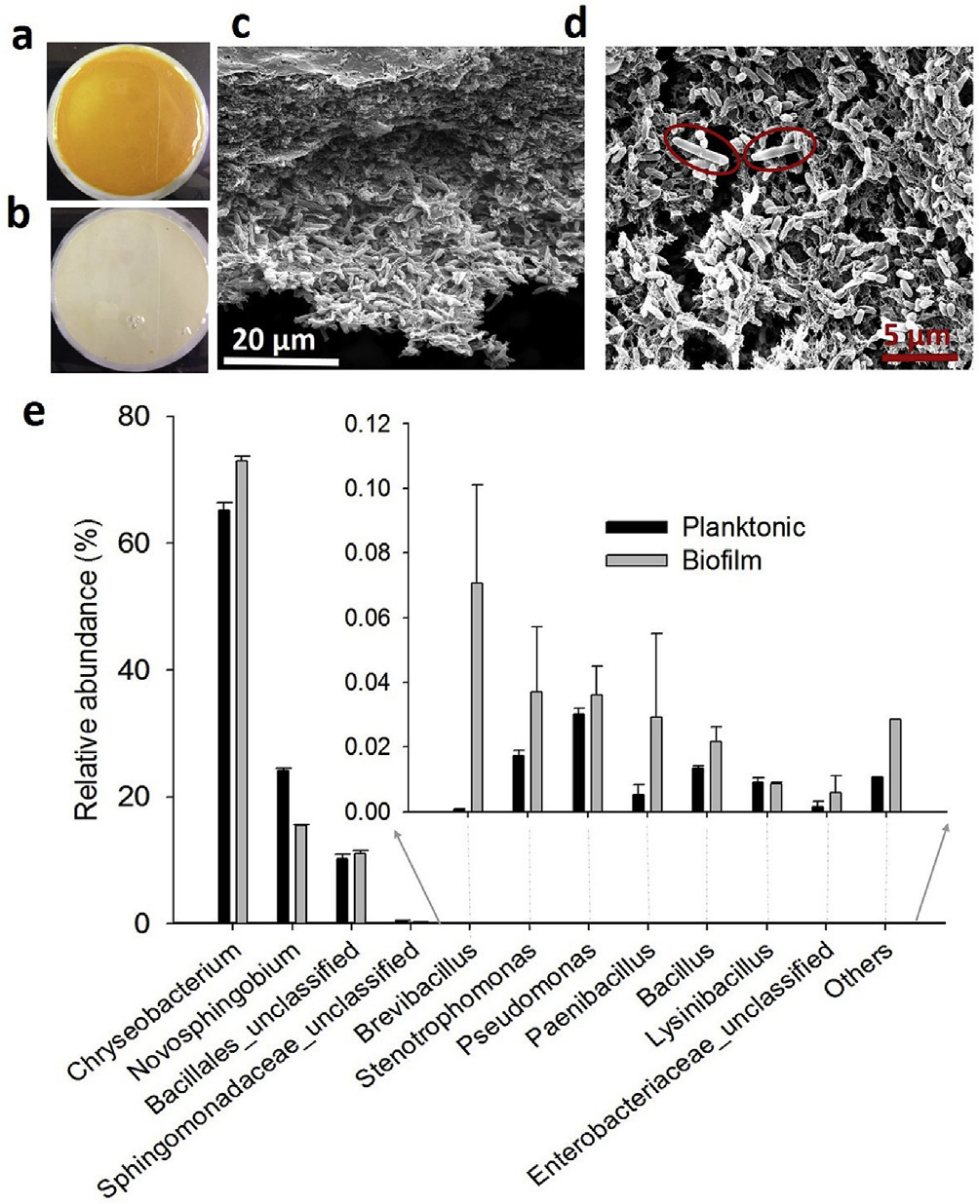
Although growth of “*Ca.* L. asiaticus” was clearly detected in the biofilms, this pathogen is still a minor member of the biofilm community. **a)** “*Ca.* L. asiaticus”-containing biofilms grown in the MBRs appear orange. **b)** Inactivated inoculum led instead to accumulation of a white precipitate. **c)** Scanning electron micrographs of “*Ca.* L. asiaticus”-containing biofilms clearly show the presence of multiple microorganism types embedded within extracellular polymeric substance. **d)** Longer, rod-shaped bacteria with the same morphology as *L. crescens* are easily detectable as a minor biofilm component. **e)** Microbial community profiles of “*Ca.* L. asiaticus”-containing biofilm and planktonic cultures revealed that 65–73% of the population of the planktonic and biofilm cultures was *Chryseobacterium* species. The error bars represent the standard error of the mean of replicated experiments (*n* = 2). Significance was determined using a *t*-test (P < 0.05). (For interpretation of the references to color in this figure legend, the reader is referred to the Web version of this article.)

The “*Ca.* L. asiaticus”-containing biofilms harvested from our MBRs were orange colored (Fig. 4a), whereas MBRs inoculated with inactivated (autoclaved) inocula only generated a white precipitate on the filter used for the MBR (Fig. 4b). All sub-cultures showed the same colors. Scanning electron micrographs of the biofilms revealed the microorganism morphotypes (Fig. 4c and d) and the presence of extracellular polymeric substance (EPS). It is possible that the EPS supports syntropy among the microorganisms in the biofilm [29]. For example, it is possible that by growing in a biofilm “*Ca.* L. asiaticus” can obtain essential nutrients from the other cells, and/or that the biofilm forms the local conditions that allow “*Ca*. L. asiaticus” to grow. Some of the bacteria grown in the biofilm had long rod shapes similar to those of *L. crescens* and “*Ca.* L. solanacearum” [30,31] (Fig. 4d, circled in red). In a previous work, Cicero et al. showed microbial biofilms containing “*Ca.* L. solanacearum” in the outer midgut surface of adult potato psyllids [32]. In addition, a recent work by Naranjo et al. demonstrated that *L. crescens* BT1 can form cell aggregates embedded in polysaccharide matrix in its pure culture, either in batch growth mode or under continuous flow [33]. These results suggest that the biofilm lifestyle may represent an adaptive advantage for the growth of “*Ca*. Liberibacter spp.” It is likely the biofilm lifestyle in MBRs allowed us to establish a host-free culture of “*Ca.* L. asiaticus.”

Metagenomic 16S rDNA sequencing showed that “*Ca.* L. asiaticus” represented a small proportion of the microbial community in the samples (∼1/100,000 reads), although PCR and qPCR were able to amplify “*Ca*. L. asiaticus” specific sequences from the cultures effectively because of their sensitivity. Quantitative estimation of the “*Ca.* L. asiaticus” 16S rDNA gene among the total 16S rDNA genes amplified from the biofilm cultures using PCR and qPCR also suggested a low fraction of “*Ca.* L. asiaticus” in its biofilm community (SI Fig. 4). Most previous reports showed very low titers of “*Ca.* L. asiaticus” in HLB-symptomatic citrus plants and infected ACPs [34,35]. Slow growth in a mixed culture under conditions that are not yet fully optimized may be a reason for the low abundance of “*Ca.* L. asiaticus” in our biofilm cultures. Further simplification of the microbial community and optimization of the medium and growth conditions may increase the proportion of “*Ca.* L. asiaticus” in MBR-grown biofilm cultures.

Metagenomic sequencing consistently identified a *Chryseobacterium* species as the most abundant biofilm community member, representing ∼65–73% of the sequencing reads (Fig. 4e), followed by a *Novosphingobium* species and an unclassified strain of *Bacillales*. These species were also found in the microbial population of extracts from “*Ca.* L. asiaticus”-infected citrus [36,37] and “*Ca.* L. asiaticus”-infected ACPs [38]. *Chryseobacterium* was previously reported in the rhizosphere microbiome of the citrus plant [37], but it has not been proposed to be associated with the development of HLB. Other minor members of the biofilm cultures (<0.1% of the sequencing reads each) were *Sphingobacterium, Bacillus, Lysinibacillus, Paenibacillus, Brevibacillus, Enterobacter* and many other genera (Fig. 4e). Most of these have been reported in the endosphere microbiome of citrus plants [37]. Note that only surface-sterilized midribs and stems from “*Ca.* L. asiaticus”*-*infected citrus plants were used to prepare the inoculum for the MBRs. Thus, the abundance of the *Chryseobacterium* species and the presence of other microorganisms of the citrus endosphere in our biofilm culture suggest their original presence inside the vascular system of the “*Ca.* L. asiaticus”*-*infected citrus plants. These microorganisms may be crucial for “*Ca.* L. asiaticus” growth *in vivo*.

We have demonstrated the feasibility of growing “*Ca.* L. asiaticus” in an axenic culture in biofilm form by inoculating from “*Ca.* L. asiaticus” -infected citrus plants. Our MBR provided specific growth conditions and a newly designed medium was used, based on a high-strength (neutral pH) buffer, additionally enhanced with nutrients including amino acids, trace minerals and vitamins. The use of the newly designed medium and MBRs allowed the growth and interaction of “*Ca.* L. asiaticus” and other microorganisms. The cultures were successfully maintained over more than two years of sequential subcultures and were generated multiple times by independent repetitions. The biofilm culture was stored and used by different researchers at a later date to reinitiate sequential cultures. This demonstrates the stability and transferability of “*Ca.* L. asiaticus”-containing biofilms cultured using our methodology. To the best of our knowledge, this is the first successful establishment of a long-term host-free culture of “*Ca.* L. asiaticus.” Host-free “*Ca.* L. asiaticus” cultures will allow researchers to examine the behavior of the pathogen in response to various factors in *in vitro* culture assays and to test treatment strategies directly (e.g., chemical treatment, nutrient and temperature controls), determining pathogen virulence. This success also opens the door to the development of a pure culture of “*Ca.* L. asiaticus” and genetics-based methods for understanding and mitigating the spread of HLB*.* Our current efforts focus on simplification of the microbial community and transitioning from a mixed-culture to a pure-culture system for *in vitro* “*Ca.* L. asiaticus” growth.

In addition to the Liberibacter spp. suspected to be the etiological agents of HLB, other Liberibacter species such as “*Ca.* Liberibacter solanacearum” [39], “*Ca.* Liberibacter psyllaurous” [40] and “*Ca.* Liberibacter europaeus” [41] are reported to be phloem-restricted pathogens that are associated with diseases in plants including potatoes, tomatoes, and pears. These pathogens share many similarities in their fastidious nature: small genome sizes with limited metabolic potential, restricted ecological niches, and slow growth [[39], [40], [41], [42]]. Analyses of their genome sequences have shown their high degree of similarity, including in predicted metabolic capacity [15,43]. Thus, the approach described here will also be helpful in culturing many other phloem-restricted microorganisms and developing strategies for curing other devastating plant diseases.

## Author contributions

P.T.H. and R.H. performed experiments. N.K. provided infected citrus trees. All authors contributed to the experimental design and to writing and editing of the manuscript.

## Competing financial interests

The authors declare no competing financial interests.
