# Supplementary material for: Host-free biofilm culture of “Candidatus Liberibacter asiaticus,” the bacterium associated with Huanglongbing
Source: Biofilm. 2019 Sep 11;1:100005. doi: 10.1016/j.bioflm.2019.100005 (PMC7798463; doi:10.1016/j.bioflm.2019.100005)
Supplement: Multimedia component 1 [file mmc1.pdf]

Host-free biofilm culture of "*Candidatus* Liberibacter asiaticus," the bacterium associated with Huanglongbing

Phuc T. Ha[1], Ruifeng He[2], Nabil Killiny[3], Judith K. Brown[4], Anders Omsland[5], David R. Gang[2] and Haluk Beyenal[1]

[1]Gene and Linda Voiland School of Chemical Engineering and Bioengineering, Washington State University, Pullman, WA 99163
[2]Institute of Biological Chemistry, Washington State University, Pullman, WA 99163
[3]Department of Plant Pathology, Citrus Research and Education Center, University of Florida, Lake Alfred, FL 33850
[4]School of Plant Sciences, University of Arizona, Tucson, AZ 85721
[5]Paul G. Allen School for Global Animal Health, Washington State University, Pullman, WA 99163

Supplementary Information

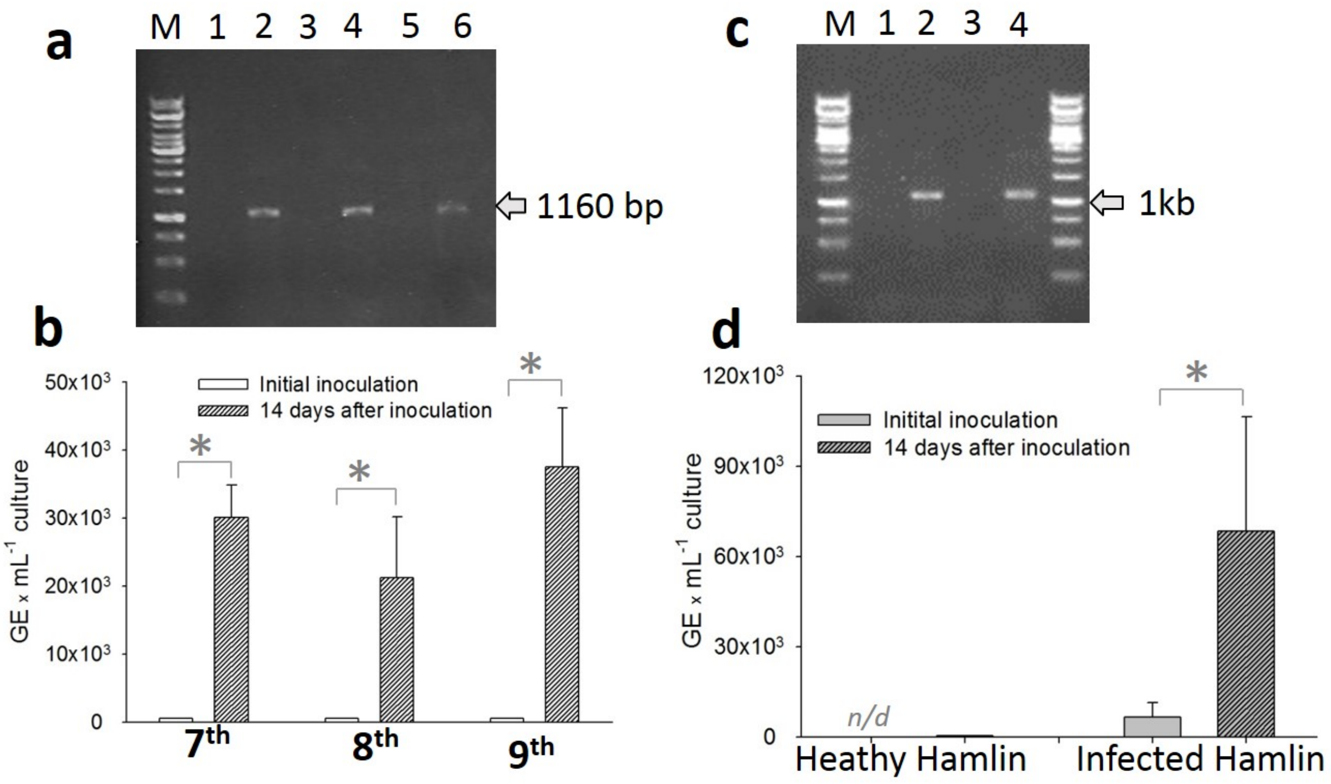

SI Figure 1. Validation of the presence and growth of "*Ca.* L. asiaticus" in biofilm cultures over transfers and independent repetitions.

a) and **b):** Validation of "*Ca.* L. asiaticus" presence and growth in the MBR at the 7th, 8th and 9th. transfers using PCR with conventional specific primers for "*Ca.* L. asiaticus" (Ol1 and Ol2c) and qPCR with a specific probe for 16S rDNA of "*Ca.* L. asiaticus." Lanes 1, 3, 5: initial inoculation; lanes 2. 4, 6: 14 days after inoculation; lane M: 1-kb marker.

c) and **d):** Validation of "*Ca.* L. asiaticus" presence and growth in a newly repeated MBR experiment in an independent laboratory. The increase of "*Ca.* L. asiaticus" genome equivalents in an MBR inoculated with a plant extract from "*Ca.* L. asiaticus"-infected Hamlin over 14 days of operation clearly demonstrates the growth of "*Ca.* L. asiaticus" in a biofilm reactor. Lanes 2 and 4: initial inoculation; lanes 1 and 3: 14 days after inoculation.

The error bars represent the standard error of the mean of replicated experiments (*n*=3).
An asterisk indicates the significant difference ((one way ANOVA-test, P<< 0.05) among the tested samples.
n/d indicates no detectable signal in qPCR.

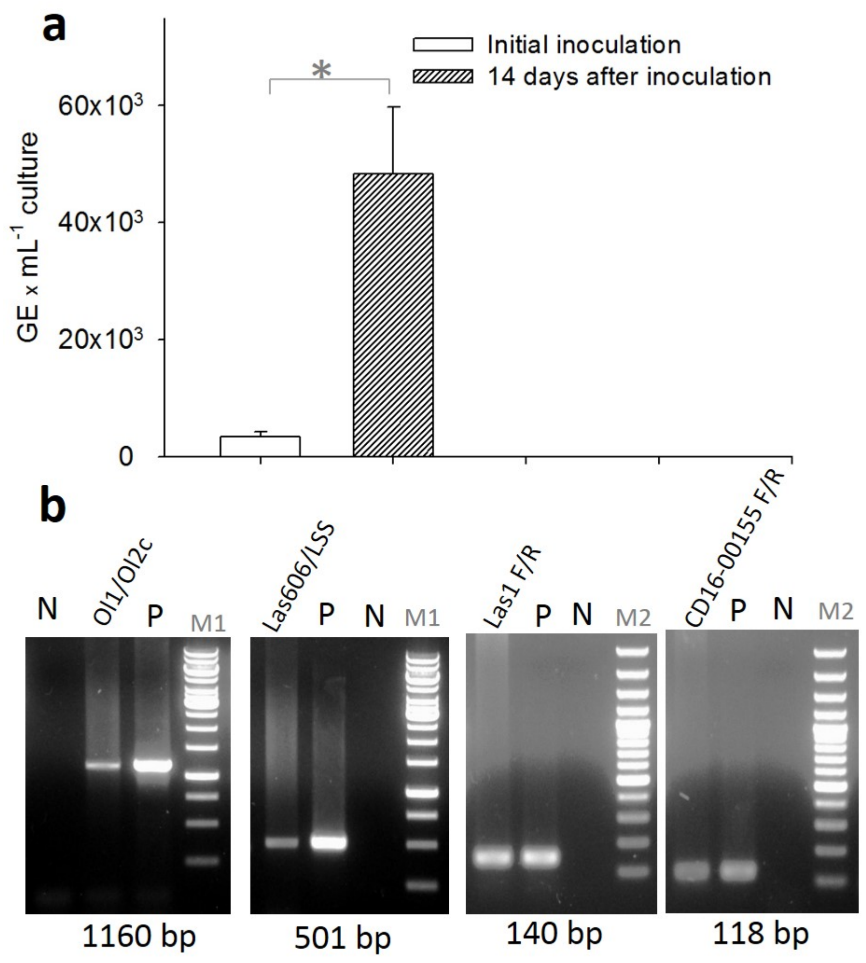

SI Figure 2. Validation of "*Ca.* L. asiaticus" presence and growth in MBR at the 12th transfer in an independent laboratory.

a) The increase of "*Ca.* L. asiaticus" genome equivalents in an MBR inoculated with 1 mL of biofilm culture from the 11th culture over 14 days of operation clearly demonstrates the growth of "*Ca.* L. asiaticus" in a biofilm reactor. The error bars represent the standard error of the mean of replicated experiments (*n*=3). An asterisk indicates the significant difference (one way ANOVA-test, P<< 0.05) among the tested samples.

b) "*C.* L. asiaticus" was detectable in PCR and qPCR assays using the following primers:

Ol1/Ol2c-1160 bp, 16S rDNA gene (PCR)

Las_606/LSS-505 bp, 16S rDNA gene (PCR)

Las1_F/R-140 bp, 16S rDNA gene (qPCR)

CD16-00155 F/R- 118 bp of hypothetical gene CD16-00155 (qPCR)

N: negative control; P: positive control; M1: marker 1 kb; M2: marker 100 bp

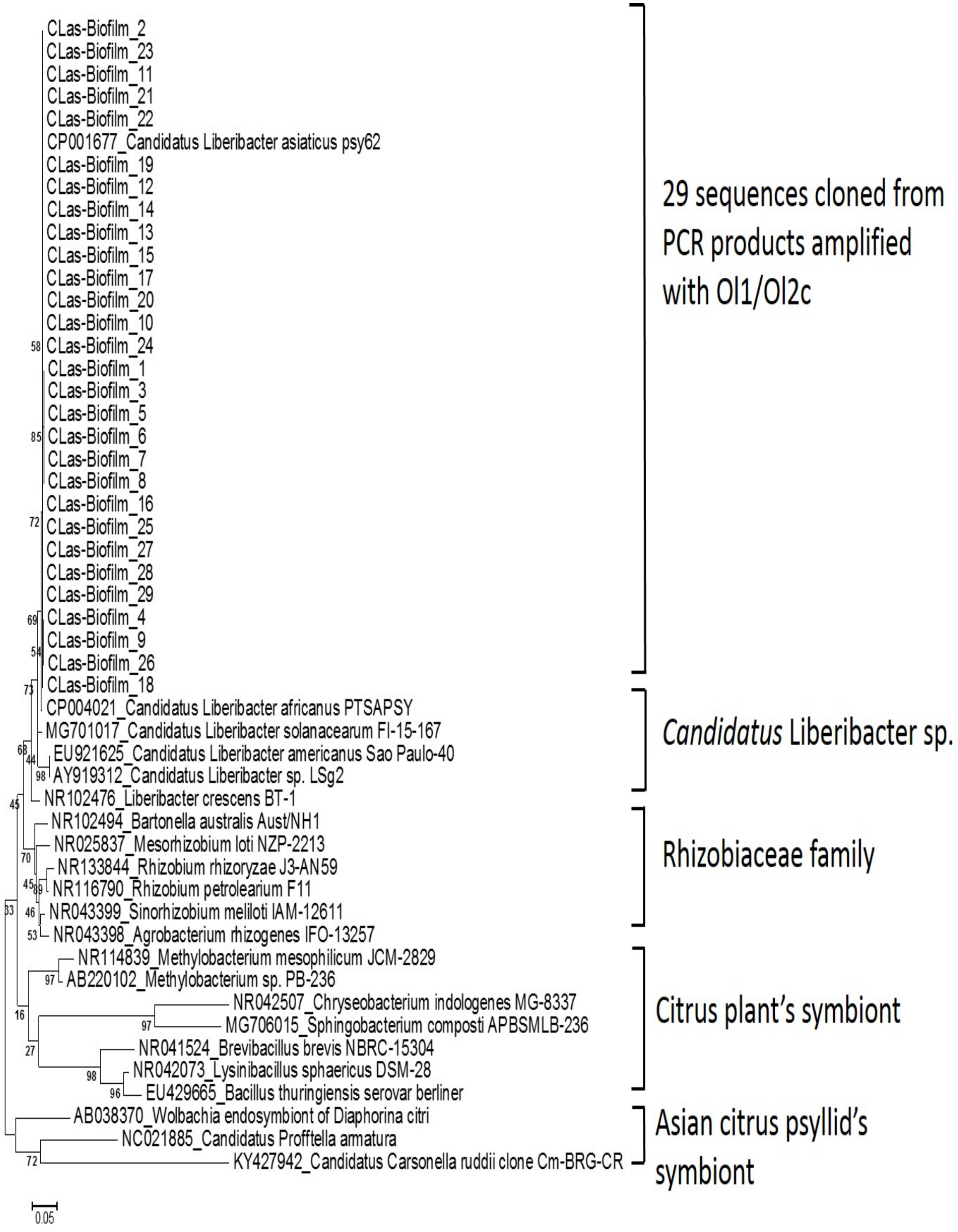

SI Figure 3. Neighbor-joining phylogenetic tree reconstructed for the cloned 16S rDNA gene fragment (1160 bp) of the cultured "*Ca*. L. asiaticus" obtained using the primers, Ol1/Ol2c, and selected "*Candidatus* Liberbacter spp.," closely-related members in the Rhizobiaceae, microbial symbionts detected in citrus plants, and three endosymbionts associated with the Asian citrus psyllid (ACP).

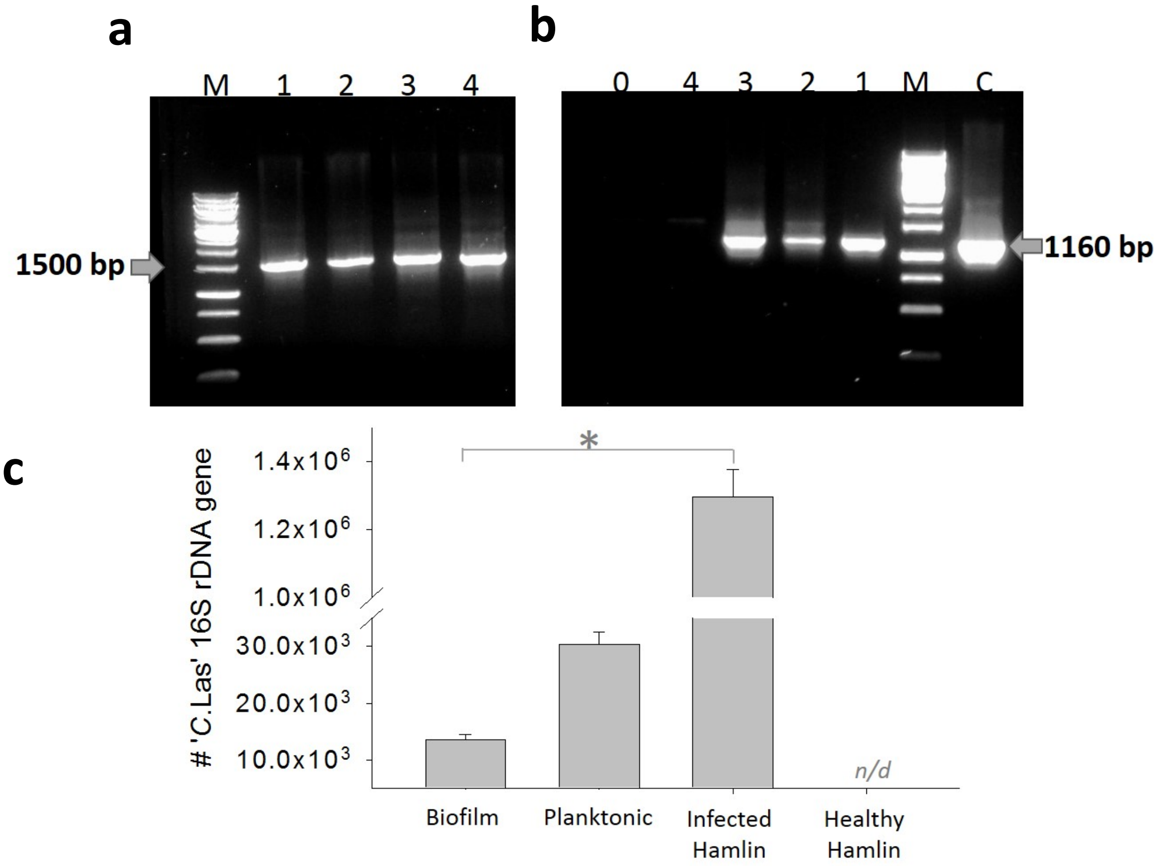

SI Figure 4. Validation and quantification of the presence of the "*Ca.* L. asiaticus" 16S rDNA gene among the total 16S rDNA genes amplified from the biofilm cultures.
(a) The total number of 1500 bp 16S rDNA gene fragments amplified from microbial communities using the universal primers, 27F/1492r. **(b)** The 16S rDNA gene fragments were purified and used as template for PCR amplification with the "*Ca.* L. asiaticus"-specific primers (Ol1/Ol2c). Amplicons of 1160 bp in size were obtained from both the biofilm cultures and from the "*Ca.* L. asiaticus"-infected citrus 'Hamlin', but not from the negative control (mockcommunity), which contained no "*Ca.* L. asiaticus". These results demonstrate the presence of the "*Ca.* L. asiaticus" 16S rDNA gene among the 16S rDNA genes amplified from other members associated with the biofilm cultures. Track 0: water; track 1 and 2: plankton and biofilm, respectively, from MBR at the 3rd transfer; track 3: HLB-symptomatic Hamlin; track 4: mock-community consisting of a mixed culture of *Staphylococcus aureus* and *Acetobacter baumannii*; track C: positive control, "*Ca.* L. asiaticus"-infected citrus. **(c)** Validation and quantification of the "*Ca.* L. asiaticus" 16S rDNA gene (C. Las's 16S rDNA gene) in 100 ng of the total number of 16S rDNA fragments of the microbial community associated with the biofilm cultures by qPCR with a "*Ca.* L. asiaticus" (Las1F/Las1R)-specific probe.

The error bars indicate the standard error of the mean of replicated experiments (*n*=3).
An asterisk indicates the significant difference ((one way ANOVA-test, P<< 0.05) among samples tested.
n/d indicates no detectable signal in qPCR.
